# Exosome-Based Delivery of Natural Products in Cancer Therapy

**DOI:** 10.3389/fcell.2021.650426

**Published:** 2021-03-02

**Authors:** Hang Song, Bin Liu, Bin Dong, Jing Xu, Hui Zhou, Sha Na, Yanyan Liu, Yunxia Pan, Fengyuan Chen, Lu Li, Jinghui Wang

**Affiliations:** ^1^Department of Biochemistry and Molecular Biology, School of Integrated Chinese and Western Medicine, Anhui University of Chinese Medicine, Hefei, China; ^2^Institute of Integrated Chinese and Western Medicine, Anhui Academy of Chinese Medicine, Hefei, China; ^3^Anhui Province Key Laboratory of Chinese Medicinal Formula, Hefei, China; ^4^Department of Cellular and Molecular Biology, Beijing Chest Hospital, Capital Medical University/Beijing Tuberculosis and Thoracic Tumor Research Institute, Beijing, China; ^5^Neurology Department, The Hefei First People’s Hospital, Hefei, China; ^6^Cancer Research Center, Beijing Chest Hospital, Capital Medical University/Beijing Tuberculosis and Thoracic Tumor Research Institute, Beijing, China

**Keywords:** exosome, natural product, cancer, therapy, delivery

## Abstract

A rapidly growing research evidence has begun to shed light on the potential application of exosome, which modulates intercellular communications. As donor cell released vesicles, exosomes could play roles as a regulator of cellular behaviors in up-taken cells, as well as a delivery carrier of drugs for targeted cells. Natural product is an invaluable drug resources and it is used widely as therapeutic agents in cancers. This review summarizes the most recent advances in exosomes as natural product delivery carriers in cancer therapy from the following aspects: composition of exosomes, biogenesis of exosomes, and its functions in cancers. The main focus is the advantages and applications of exosomes for drug delivery in cancer therapy. This review also summarizes the isolation and application of exosomes as delivery carriers of natural products in cancer therapy. The recent progress and challenges of using exosomes as drug delivery vehicles for five representative anti-cancer natural products including paclitaxel, curcumin, doxorubicin, celastrol, and β-Elemene. Based on the discussion on the current knowledge about exosomes as delivery vehicles for drugs and natural compounds to the targeted site, this review delineates the landscape of the recent research, challenges, trends and prospects in exosomes as delivery vehicles for drugs and natural compounds for cancer treatment.

## Introduction

Cancer is one of the major treats to human life worldwide. In the Western world, the mortality of cancer has decreased, but cancer mortality remains high in the developing and underdeveloped countries. In 2012, 64.9% of cancer-related deaths occurred in underdeveloped regions (Ferlay et al., 2013). The cost of cancer care is high, which limits proper cancer treatment. In recent years, natural products have been proven to have various anti-cancer properties, including inhibiting cell proliferation, inducing cell apoptosis or autophagy, interfering with cancer angiogenesis, invasion or metastasis, and modulating epigenetic modifications (Wang et al., 2010; Zhuang et al., 2012). Using natural products for cancer management is an appealing alternative to overcome expensive cancer care, especially in developing or underdeveloped countries.

Numerous studies have shown that natural products have poor solubility, rapid biotransformation and low bioavailability *in vivo*, that limit their pharmacological activities (Brglez Mojzer et al., 2016; Huang et al., 2018; Zhang et al., 2019). For example, cuicumin, one of the natural products shown to have multiple-pharmacological roles, is reported to have a low plasma concentration, extensive and rapid biotransformation, and poor oral bioavailability (Huang et al., 2018). Magnolol, a hydroxylated biphenyl natural compound, was reported to have multiple-pharmacological characteristics including anti-inflammatory, anti-microorganism, anti-oxidative, anti-cancer, neuroprotective, and cardiovascular protective effects. Yet, it also has low water solubility, low bioavailability, and rapid metabolism (Zhang et al., 2019). Polyphenols, as secondary plant metabolites, are reported to have many advantages for anti-cancer effects such as high accessibility, low toxicity, and specificity of response, but have limited usage in clinics because of their poor bioavailability and rapid metabolism (Brglez Mojzer et al., 2016). Therefore, it would be useful to find a new drug delivery system to improve the bioavailability of natural products *in vivo*.

Nanotechnology has been employed for drug delivery for increasing bioavailability of therapeutic agents. Unfortunately, drug nanoformulations often lead to toxicity and are usually rapidly cleared by the mononuclear phagocytic system (MPS) (Peng et al., 2013). Although PEGylation of drug-loaded nanocarriers could reduce the clearance by the MPS, it reduces the biodistribution of drug in disease tissues (Veronese et al., 2002). Moreover, rapid generation of anti-PEG antibodies following repeated injections of PEGylated nanoparticles would result in extended blood clearance and decreased efficacy of nanoformulations (Gabizon, 2001). Furthermore, biological barriers reduce the bioavailability and limit the therapeutic efficacy of nanoformulations (Blanco et al., 2015). Therefore, new targeted deliveries of drugs should be studied to avoid the clearance and overcome the biological barriers.

Exosomes have emerged as drug delivery vehicles. Exosomes deliver nucleic acids (Pan et al., 2012; Wahlgren et al., 2012), proteins (Haney et al., 2015), and small molecule drugs, such as doxorubicin (Tian et al., 2014). As delivery vehicles, exosomes deliver their payload to target cells or tissues, and diminish the MPS-mediated clearance (Wiklander et al., 2015). Moreover, siRNA could be delivered across the blood-brain barrier by exosomes to the central nervous system (Alvarez-Erviti et al., 2011). These results demonstrate that exosomes may be a promising alternative to nanoparticles as drug delivery vehicles (Parodi et al., 2017). The focus of this review is the anti-cancer application of natural products delivered by exosomes.

## The Composition of Exosomes

Chargaff and West (1946) reported that plasma clotting was inhibited by the removal of the pelleted plasma fraction. Subsequently, Wolf (1967) reported that these clotting suppressors are vesicles in the range of 20–50 nm secreted by platelets. Since then, a number of studies have indicated the existence of extracellular vesicles. Extracellular vesicles include three forms: exosomes, microvesicles, and apoptotic bodies (Gyorgy et al., 2011). Exosomes are 30–150 nm in diameter, and are secreted by various kinds of cells including dendritic cells (Thery et al., 2006), macrophages (Bhatnagar et al., 2007), B cells (Clayton et al., 2005), T cells (Nolte-’t Hoen et al., 2009), mesenchymal stem cells (Lai et al., 2015), endothelial cells (Song et al., 2014), and epithelial cells (Skogberg et al., 2015), and a variety of cancer cells (Benito-Martin et al., 2015).

**FIGURE 1:**
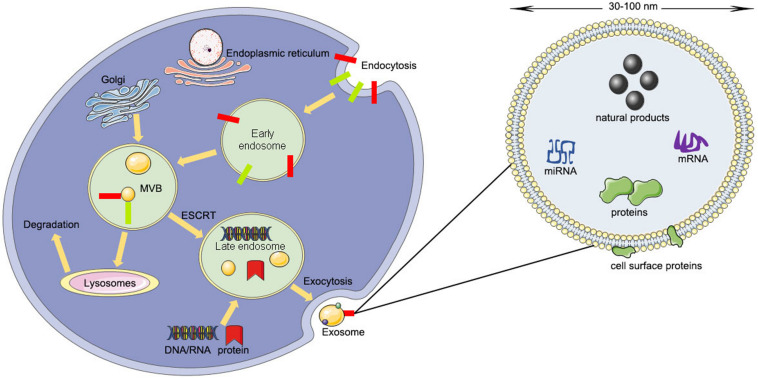
The producing process and structural composition of exosomes (ESCRT, endosomal-sorting complex required for transport; MVB, multivesicular body).

The contents of exosomes include lipids, nucleic acids, and various proteins such as receptors, enzymes, transcription factors, and extracellular matrix proteins, that are inside or on the surface of exosomes (D’Asti et al., 2012). The lipid content is cell-specific or conserved and can protect the shape of exosomes, joins in the biogenesis of exosomes, and regulates the homeostasis of the recipient cells (Minciacchi et al., 2015). For example, lysobisphosphatidic acid (LBPA) was reported to interact with Alix regulating the invagination of the endosomal membrane (Laulagnier et al., 2004) and result in the formation of exosomes (Chu et al., 2005; Bissig et al., 2013). However, the protein contents of exosomes can be divided into a specific type and a non-specific type (Van Niel et al., 2006). The specific type of proteins include integrins, tetraspanins, adhesion molecules, transferrin receptors, and major histocompatibility complex (MHC) class I and II (Van Niel et al., 2006). The non-specific type of proteins include transferring proteins and fusion, cytoskeleton proteins, and heat shock proteins (Van Niel et al., 2006; Poliakov et al., 2009) (Figure 1).

These contents of exosomes can reflect the composition of the donor cell and the mechanism of physiological or pathological changes (Liu and Pilarsky, 2018). For example, antigen-presenting cells secrete the exosomes carrying T cell co-stimulatory molecules, MHC class I and class II molecules on the surface, that play important roles in antigen presentation (Schorey et al., 2015). Endothelial cells secrete exosomes containing high levels of DLL4 (delta-like-4) protein, which can activate the Notch signaling pathway and induce capillary sprouts in the neighboring microvascular endothelial cells (Sharghi-Namini et al., 2014). And miR-222 from tumor-derived exosomes can down-regulate the level of Pdlim2 resulting in enhanced metastatic capacity in breast cancer cells (Ding et al., 2018).

## The Biogenesis of Exosomes

Unlike microvesicles budding directly from the plasma membrane, exosomes arise from the invagination of the endosomal membrane (Simons and Raposo, 2009). The first step is the fusion of primary endocytic vesicles forming early endosomes (EEs) (Huotari and Helenius, 2011). EE can either return the cargo to the plasma membrane or change into “late endosomes” (LEs) by inward budding of the membrane with the cargo packed (Mashouri et al., 2019).

The package of proteins into the intraluminal vesicles is dependent on the ESCRT (endosomal-sorting complex required for transport), which includes four complexes: ESCRT-0, ESCRT-I, ESCRT-II, and ESCRT-III (Mashouri et al., 2019). ESCRT-0 recognizes mono-ubiquitinated proteins with the help of an HRS heterodimer, which recruits clathrin to help ESCRT-0 encounter the ubiquitinated cargo (Ren and Hurley, 2010). Next, ESCRT-I, ESCRT-II, and ESCRT-0 constitute a recognition domain of ubiquitinated substrates (McGough and Vincent, 2016). Subsequently, ESCRT-III joins the complex, pinches off the membrane, and releases the buds into the endosome (Wollert et al., 2009). Then the intraluminal vesicles will be degraded within the lysosome unless de-ubiquitylating enzymes (DUBs) de-ubiquitinated the cargoes (Yeates and Tesco, 2016). The intraluminal vesicles can be released into the extracellular environment by moving to the plasma membrane (Kumar et al., 2016). Rab27A and Rab27B are the crucial mediators to lead the vesicles toward the cell periphery (Ostrowski et al., 2010). Finally, the membrane fusion and exosome secretion are completed by the soluble N-ethylmaleimide (NEM)-sensitive factor attachment protein receptor (SNARE) complex (Kennedy and Ehlers, 2011).

Sometimes the package of proteins into the intraluminal vesicles is carried out by the ESCRT-independent pathway. The ESCRT-independent mechanism occurs in the melanosome of melanocytes. Pmel17 is the crucial mediator in the formation of the intraluminal vesicles in an ESCRT-independent manner, which can connect its luminal domains with lipids (Theos et al., 2006). Tetraspanin CD63 is another mediator for the invagination of the melanosome membrane in an ESCRT-independent manner (Theos et al., 2006; Van Niel et al., 2011). Moreover, proteolipid proteins are delivered from the endosomal membrane to the intraluminal vesicles in an ESCRT-independent manner, which might suppress the formation of the intraluminal vesicles (McGough and Vincent, 2016).

## The Functions of Exosomes in Cancer

**FIGURE 2:**
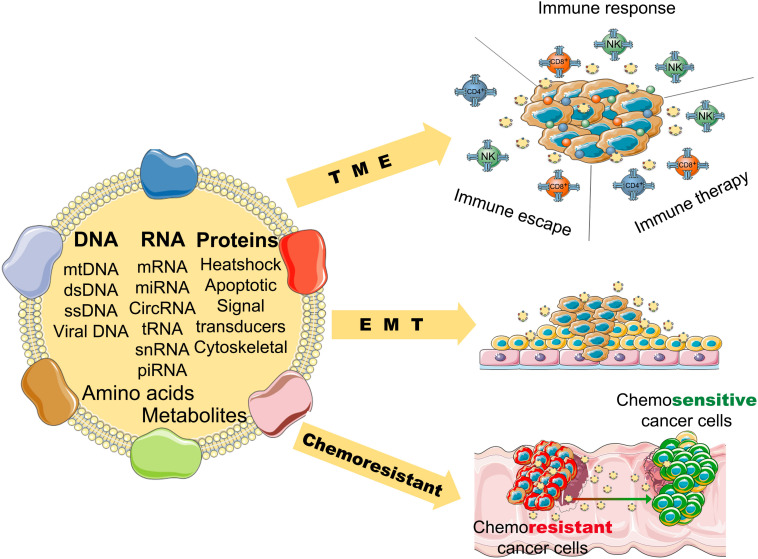
The potential role of tumor-derived exosomes in the progression and pathological process of cancer (TME, tumor microenvironment; EMT, epithelial-mesenchymal transition).

Numerous studies reveal that exosomes have a wide variety of functions in cancers. First, tumor microenvironment (TME), endothelial cells, fibroblasts, and infiltrating immune cells interact with tumor cells, and these interactions are determined by the contents of the exosomes (Kohlhapp et al., 2015). Exosomes also activate the extracellular receptor signals and block cell adhesion to modulate the TME and extracellular matrix (Luga et al., 2012; Sung et al., 2015). For example, exosomal integrins take part in the initial colonization of cancer cells and the formation of a pre-metastatic niche (Paolillo and Schinelli, 2017). Exosomal miR-105 can downregulate the level of ZO-1 and destroy the barrier function of endothelial monolayers, resulting in metastasis and vascular permeability in distant organs (Zhou et al., 2014). Exosomes from cancer cells can induce differentiation of TME cells to cancer-associated fibroblasts (CAFs), that are the dominant cell population of the TME in most cancers (Webber et al., 2010) (Figure 2).

Second, exosomes can promote angiogenesis and induce EMT (epithelial to mesenchymal transition) (Syn et al., 2016), that favor the motility and dissemination of tumor cells. Exosomes are associated with one of the main mechanisms resulting in angiogenesis. Exosomes carry many kinds of angiogenic stimulatory factors such as VEGF (vascular endothelial growth factor), PDGF (platelet-derived growth factor), TGF-β (transforming growth factor β), and bFGF (basic fibroblast growth factor) (Katoh, 2013). Exosomes also induce reprogramming and modulation of endothelial cells to promote angiogenesis (Ludwig et al., 2018). Furthermore, it has been reported that exosomes in pivotal position contribute to all process of EMT, form invasive phenotype to distant metastasis (Whiteside, 2017). Matrix metalloproteinase (MMP) 13-loading exosomes promote metastasis by inducing EMT in nasopharyngeal cancer cells (You et al., 2015). Exosomes derived from bladder cancer cells can promote EMT in urothelial cells by increasing the expressions of mesenchymal biomarkers, such as α-SMA, S100A4, and snail, and decreasing the expressions of epithelial biomarkers, including E-cadherin and β-catenin (Franzen et al., 2015).

Moreover, exosomes play an important role in the chemoresistance of cancers. Tumor cells can pack the chemotherapeutic drugs into exosomes and shuttle them out (Safaei et al., 2005). The contents carried by exosomes are associated with tumor drug resistance (Shedden et al., 2003). For example, miR-155 delivered by exosomes can increase EMT biomarkers to induce chemoresistance in breast cancer cells (Santos et al., 2018); miR-32-5p delivered by exosomes can cause multi-drug resistance by promoting angiogenesis and EMT (Fu et al., 2018). Tumor-derived exosomes can inhibit the response of immune effector cells and induce immune suppressor cells to modulate the TME, which results in chemoresistance of cancers (Hellwinkel et al., 2016; Syn et al., 2016). And exosomes can use a decoy to help cancer cells evade the immune effector cells (Battke et al., 2011).

## The Advantages and Applications of Exosomes for Drug Delivery in Cancer Therapy

Although exosomes have the capacity to promote the progression of cancers, exosomes show advantages in drug-delivery because of their good biodistribution, biocompatibility, and low immunogenicity. Exosomes have good tolerance because of their similarity to the cell membrane in structure and composition (Bang and Thum, 2012). Some exosomes can evade the immune system (Hood, 2016). For example, Adriamycin-loaded exosomes have minimal immunogenicity and toxicity (Tian et al., 2014). Comparison with liposomes, exosomes permeate tumor cells with higher rate (Kohlhapp et al., 2015). Because exosomes are small, they can pass through bodily barriers. In 2011, some studies indicated the feasibility of using exosomes for drug delivery for the first time by delivering siRNA across the blood-brain barrier (BBB) using exosomes derived from dendritic cells (Alvarez-Erviti et al., 2011). Also, exosomes could promote targeting efficiency of anti-cancer drugs with easy manipulation (Li et al., 2017).

Recently, the applications of exosomes in the delivery of chemotherapeutic drugs have exhibited enhanced curative effects in cancer therapy. For example, paclitaxel-loaded exosomes can be used to treat prostate, lung, and pancreatic cancers (Saari et al., 2015). Doxorubicin-loaded exosomes also showed great efficiency in breast cancer cells (Tian et al., 2014). However, exosomes from different donor cells play different physiological functions. For example, tumor-derived exosomes can play a role of anti-tumor immunity by carrying tumor-specific antigens, proteins and miRNAs, but they can induce apoptosis of T cells, inhibit monocyte differentiation, and induce a pro-inflammatory microenvironment (Taylor and Gercel-Taylor, 2011). Exosomes from mesenchymal stem cells can regulate immunity and promote tissue repair, but they can promote tumor growth by activating tumor angiogenesis related factors (Zhu et al., 2012). Exosomes from immune cells can avoid the clearance of the immune system and prolong the retention time in the peripheral circulation (Haney et al., 2015). Milk-derived exosomes have no immune exclusion and inflammatory reaction and can improve the oral bioavailability of drugs (Ju et al., 2013). Therefore, it is necessary to select exosomes derived from appropriate donor cells when selecting exosomes for drug-delivery.

## The Isolation and Application of Exosomes in the Delivery of Natural Products in Cancer Therapy

At present, there are many methods to isolate exosomes from bodily fluids or conditioned cell culture media, such as filtration paired with centrifugation, immunoaffinity chromatography, size exclusion chromatography, polymer-based precipitation, differential centrifugation, and microfluidic technologies (Witwer et al., 2013). Among these methods, differential ultracentrifugation and density gradient centrifugation are considered to be the “gold standard” methods (Thery et al., 2006). Each method has its two sides, advantages and disadvantages, and which method is selected is dependent on the user’s application. The combination of different methods can maximize advantages and avoid disadvantages compared to a single method (Stremersch et al., 2016).

**FIGURE 3:**
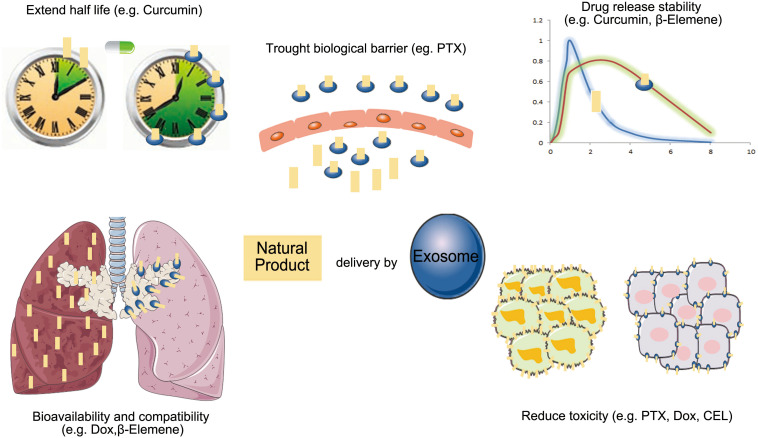
The advantage of exosomes carrier natural products in anti-cancer therapy (PTX, paclitaxel; Dox, doxorubicin; CEL, celastrol).

Because exosomes as drug delivery carriers have good biodistribution, biocompatibility, and low immunogenicity, more researchers have begun to study their applications for enhancing the bioavailability of natural products in cancer therapy (Figure 3).

### Paclitaxel (PTX)

PTX is a microtubule-stabilizing agent that exhibits anticancer effects in many malignant tumors, such as glioblastoma multiforme (GBM) tumors (Salarpour et al., 2019) and breast cancer (Agrawal et al., 2017). Cisplatin-resistant cancer patients often retain sensitivity to PTX (Aqil et al., 2017a). However, some studies report that PTX has low bioavailability and cannot pass through BBB (Xin et al., 2012; Mu et al., 2015). It was reported that PTX has a dose-dependent toxic effect, which hamper the application of PTX in clinical trials (Wang et al., 2019).

Research from Italy firstly presented that mesenchymal stromal cells could package PTX into exosomes and exhibit enhanced anticancer effects of PTX (Pascucci et al., 2014). Recently, various studies demonstrated that exosomes used as PTX carriers could enhance the anticancer effects of PTX. American scientist reported that milk-derived exosomes for oral delivery of PTX showed better tumor suppressor properties against human lung tumor xenografts in nude mice, and had lower systemic and immunologic toxicities as compared to i.v. PTX (Agrawal et al., 2017). Some studies reported that exosomes from M1-polarized macrophages enhanced the antitumor effect of PTX by activating macrophage-mediated inflammation in tumor-bearing mice (Wang et al., 2019). It was reported that exosomes from U-87 cells could pass through BBB and enhanced the anticancer effects of PTX in GBM (Salarpour et al., 2019). Another study indicated that embryonic stem cell-derived exosomes could improve the curative effect of PTX via enhanced targeting in GBM (Zhu et al., 2019). American researchers reported that macrophage-derived exosomes could enhance the antitumor effect of PTX in resistant cancer cells (Kim et al., 2016). They further reported that the aminoethylanisamide-polyethylene glycol-vectorized exosomes derived from macrophages possessed a high loading capacity of PTX, an enhanced ability to accumulate in cancer cells upon systemic administration, and better therapeutic outcomes (Kim et al., 2018). Moreover, it was reported that cancer cell-derived exosomes showed potential carrying capacity of PTX to their parental cells. They may bring the drug into the target cells by endocytic pathway to achieve high cytotoxicity (Saari et al., 2015).

### Curcumin

Curcumin as a natural polyphenol compound can mitigate the initiation and metastasis of pancreatic, colon, breast, oral, and several other cancers (Ramayanti et al., 2018). Several clinical trials for the treatments of cancers have addressed the safety, pharmacokinetics, and efficacy of using curcumin in humans (Dhillon et al., 2008). The dominant features, inexpensive and low toxicity made curcumin ideal for clinical applications (Chen et al., 2012). However, curcumin has low bioavailability, low solubility in water, short half-life in plasma, and low stability (Salehiabar et al., 2018), which limits its usage in patients.

Previous studies showed that exosomes could enhance the anti-inflammatory activity of curcumin, and the formation of exosome-curcumin complexes could increase the stability of curcumin *in vitro* and its bioavailability *in vivo* (Sun et al., 2010). Scientist used exosomes to encapsulate curcumin and gave the exosomes-curcumin complex to a GL26 brain tumor model via an intranasal route, which significantly delayed brain tumor growth with reduced inflammation and mitigated the dysfunction of the brain endothelial cells (Zhuang et al., 2011). Previous research indicated that although exosomes derived from pancreatic cancer cells increased the proliferation of pancreatic cancer cells, curcumin-loaded exosomes induced the apoptosis of pancreatic cancer cells (Osterman et al., 2015). American scientists reported that milk-derived exosomes could enhance the antitumor activity of curcumin both *in vitro* and *in vivo* without gross or systemic toxicity (Aqil et al., 2017b). A recent study supported that both cow milk-derived and intestinal epithelial cell-derived exosomes could improve cellular uptake and intestinal permeability of curcumin, that confirm the bioavailability of an oral drug can be enhanced by the exosomes-based delivery (Carobolante et al., 2020). Furthermore, Chinese scientists loaded curcumin into exosomes, and conjugated the exosome membrane with neuropilin-1-targeted peptide to obtain glioma-targeting exosomes. These exosomes smoothly crossed the BBB and provided good results for targeted imaging and therapy of glioma (Jia et al., 2018). It has been reported that exosomes loaded with curcumin could increase the levels of claudin-5, occludin, ZO-1, and VE-cadherin, that played important roles in the integrity of cerebral tight junctions and adherent junctions (Kalani et al., 2014). Exosomes loaded with curcumin could attenuate the toxicity induced by homocysteine, a compound capable of disrupting the BBB (Kalani et al., 2014).

### Doxorubicin (Dox)

Dox is one of the most effective anticancer agents and is used in a wide variety of cancers including solid tumors, transplantable leukemia, and lymphomas. However, the clinical usage of Dox is limited because of its low bioavailability and severe side effects, such as bone marrow suppression and cardiotoxicity. Although nanoparticles have been used as deliveries of Dox to increase its anti-tumor effects, nanoparticles can cause adverse effects such as immune responses and oxidative stress (Yang et al., 2015).

Recently, exosomes as natural nanoparticles have been studied to deliver Dox. Studies proved that exosomes from mesenchymal stem cells could enhance cellular uptake efficiency and anti-tumor effects of Dox in osteosarcoma MG63 cells (Wei et al., 2019). Scientists designed targeted exosomes from mesenchymal stem cells with a chimeric protein against HER2-positive breast cancer, which was used to deliver Dox to HER2-positive cancer cells, resulting in the selective distribution and enhanced antitumor effect of Dox (Gomari et al., 2019). Furthermore, researcher form China designed exosomes with disintegrin and metalloproteinase 15 expressing on exosomal membranes, and packed Dox and cholesterol-modified miRNA 159 into the modified exosomes, resulting in improved anticancer effect of Dox without adverse effects (Gong et al., 2019).

### Celastrol (CEL)

CEL is a plant-derived triterpenoid and has anticancer effect against a wide variety of cancers (Li et al., 2018, 2019; Jiang et al., 2019). It can induce apoptosis of vincristine-multidrug-resistant oral cancer cells via JNK1/2 signaling pathway (Lin et al., 2019). However, due to its poor bioavailability and off-site toxicity, the clinical usage of CEL is limited (Freag et al., 2018).

CEL was packed into exosomes derived from milk and the effect of CEL-loading exosomes on lung cancer cells was studied (Aqil et al., 2016). It was found that exosomes enhanced the anticancer effects of CEL on lung cancer *in vitro* (Aqil et al., 2016). CEL-loading exosomes are stable and could be delivered orally, exhibiting enhanced biological efficacy without gross or systemic toxicity *in vivo* (Aqil et al., 2016).

### β-Elemene

β-Elemene, a natural compound extracted from Zedoary, has effects against a wide variety of tumors (Zhang et al., 2014; Gong et al., 2015; Jiang et al., 2017). It can reverse multidrug resistance and increase the sensitivity of chemotherapeutic drugs (Guo et al., 2014).

There are studies showed that β-elemene could promote the release of exosome to inhibit the growth of lung cancer cells, demonstrating that exosomes are involved in the anticancer effects of β-elemene (Li et al., 2014). Researchers used β-elemene-loaded exosomes to treat drug-resistant breast cancer cells, and found that β-elemene-loaded exosomes reverse the drug-resistance of breast cancer by down-regulating the expression of P-gp (Zhang et al., 2015).

Natural compounds can modify the contents of exosomes. For example, docosahexaenoic acid (DHA) can promote the secretion of exosomes and increase the levels of small RNA in the exosomes to inhibit pro-angiogenetic mRNAs, resulting in the suppression of tumor angiogenesis in breast cancer cells (Hannafon et al., 2015). Tea polyphenol epigallocatechin gallate (EGCG) can up-regulate miR-16 in the exosomes from murine breast cancer cells, resulting in decreased levels of CSF-1 and CCL2, two growth factors associated with tumor promoting associated macrophages (M2) (Jang et al., 2013).

## Conclusion

Although the use of natural products can reduce the cost of cancer care, the applications are limited because of their poor solubility, rapid biotransformation, and low bioavailability. For improving the therapeutic index of natural products, their delivery should be improved. Conventional drug delivery has some disadvantages including low therapeutic index and adverse side effects. Various biological barriers prevent drugs from reaching the tumor site with an efficacious therapeutic dose. Efficient delivery of natural products should have these features, including circulation in the bloodstream without opsonization, escaping surveillance of the immune system, preserving their contents, delivering a drug into the targeted site of tissues, overcoming the biological barriers, penetrating the membranes of target cells, and minimizing accumulation at undesired sites. There are many progress in drug delivery by nanotechnology. Unfortunately, nanoparticles have some disadvantages, such as toxicity and rapid clearance. Exosomes are a promising alternative to nanoparticles because of their advantages in drug delivery, such as a high drug-carrying capacity, non-cytotoxic effects, and a low immunogenic profile. Exosomes can prolong time of circulation in the blood, reduce the levels of clearance, and protect contents from degradation or inactivation. However, the technological, functional and safety features of exosome-based drug formulations need to be further elucidated. Deficiencies in our knowledge for the molecular mechanisms of exosome biogenesis, and no method to interfere with the package of contents or with vesicle release, still hampers the identification of their physiological relevance *in vivo*.

It is a meaningful and feasible way to explore the exosome-like vesicles for delivering natural products in targeting and penetrating solid tumor with effectively therapeutic doses in clinical cancer therapy. In this review, we summarized the advantages of exosomes and showed that exosomes offer new possibilities for cancer treatment, potentially as drug delivery vehicles for the natural products. We also discuss the problems in the research of exosomes. However, exosome will still be an attractive method for delivering the natural products in the cancer treatments.

## Author Contributions

HS, BL, and JW participated in the design of this review and revised manuscript. BL, BD, HS, and LL wrote the manuscript. JX, HZ, SN, YL, YP, and FC collected literature and made a preliminary summary. All authors contributed to the article and approved the submitted version.

## Conflict of Interest

The authors declare that the research was conducted in the absence of any commercial or financial relationships that could be construed as a potential conflict of interest.
